# Differential Growth Responses of *Alternanthera philoxeroides* as Affected by Submergence Depths

**DOI:** 10.3389/fpls.2022.883800

**Published:** 2022-06-02

**Authors:** Shufang Jing, Xiaoping Zhang, Hangang Niu, Feng Lin, Qiaoli Ayi, Binna Wan, Xinyi Ren, Xiaolei Su, Shaohua Shi, Songping Liu, Bo Zeng

**Affiliations:** ^1^Key Laboratory of Eco-environments in Three Gorges Reservoir Region (Ministry of Education), Chongqing Key Laboratory of Plant Ecology and Resources in Three Gorges Reservoir Region, School of Life Sciences, Southwest University, Chongqing, China; ^2^School of Art and Design, Huanghuai University, Zhumadian, China

**Keywords:** adaptive strategy, submergence-tolerant plants, hydrostatic pressure, flood-prone habitats, submergence depths

## Abstract

Global climate change has resulted in an increase in intensity and frequency of flooding, plants living in lowlands, and shore areas have to confront submergence caused by flooding, submergence-tolerant plants usually respond by adopting either escape or quiescence strategies. While certain plants exhibit a changeover from escape strategy upon partial submergence to quiescence strategy under complete shallow submergence, it remains unknown whether plants completely submerged at different water depths would adjust their strategies to cope with the change in submergence depth. *Alternanthera philoxeroides* is an ideal species to explore this adjustment as it is widely distributed in flood-disturbed habitats and exhibits an escape strategy when completely submerged in shallow waters. We investigated the responses of *A. philoxeroides* in terms of morphology, anatomy, and non-structural carbohydrate metabolism by conducting experiments using a series of submergence depths (0, 2, 5, and 9 m). During the submergence treatment, environmental factors such as light, dissolved oxygen, and temperature for submerged plants were kept constant. The results showed that *A. philoxeroides* plants submerged at depth of 2 m presented an escape strategy *via* fast stem elongation, extensive pith cavity development, and small biomass loss. However, the retarded stem elongation, reduced pith cavity transverse area, and increased biomass loss along the water depth gradient indicated that *A. philoxeroides* altered its growth response as water depth increased from 2 to 9 m. It is found that the changeover of response strategies occurred at higher submergence depths (5–9 m). Based on the results of our experiments, we demonstrated that water depth played an important role in driving the change in strategy. The water-depth-dependent growth performance of *A. philoxeroides* would benefit the species in habit exploration and exploitation. Further studies should focus on the performances of plants when submerged at varied water depths with different light climates and dissolved oxygen content, and how water depths drive the response behaviors of the submerged plants.

## Introduction

Global climate change has increased the intensity and frequency of flood events, and this trend is predicted to continue in the future (Milly et al., 2002; Blöschl et al., 2019). River engineering has considerably altered the hydrological regimes of rivers worldwide (Dynesius and Nilsson, 1994; Huang et al., 2016). These changes mainly include the transition from shallow floods to deep floods (Belt, 1975; Brock et al., 1987; Sparks et al., 1998; Bertola et al., 2020). The construction of more than 50,000 dams higher than 15 m worldwide (Lehner et al., 2011) has resulted in huge drawdown zones. For example, the Three Gorges Reservoir in China has a drawdown zone of ~350 km^2^ with a maximum submergence depth of 30 m, the water level of which fluctuates annually between 145 and 175 m above sea level (Lei et al., 2017). Such deep submergence has detrimental effects on the growth and survival of plants (Lei et al., 2014; Bejarano et al., 2018; Huang et al., 2021), especially on those completely submerged (Vervuren et al., 2003; Fukao et al., 2019; Striker et al., 2019). As a consequence, plants have to strive to cope with submergence in flood-prone habitats.

One of the major problems that terrestrial plants face when submerged is the energy crisis, induced by low gas partial pressure and light intensity in water (Bailey-Serres et al., 2012; Huber et al., 2012; Pedersen et al., 2017). Gas (e.g., oxygen, carbon dioxide, and ethylene) exchange between completely submerged plants and water column significantly slows as gas diffusion is severely restricted underwater (Jackson, 1985; Voesenek et al., 2006; Voesenek and Bailey-Serres, 2015). Due to the low oxygen content in the water column, the aerobic respiration of completely submerged plants decreases, leading to reduced ATP production and even plant death (Voesenek et al., 2006; Bailey-Serres and Voesenek, 2008). Compared to the atmosphere, water bodies have relatively low levels of CO_2_, moreover, the photosynthetically active radiation (PAR) in water bodies sharply declines from the water surface downwards (Voesenek et al., 2006; Voesenek and Bailey-Serres, 2015). This further limits underwater photosynthesis and increases the energy crisis of plants when submerged.

The escape and quiescence strategies are two major strategies adopted by plants in flood disturbed habitats (van Veen et al., 2014). Generally, the escape strategy enables plants to cope with shallow but prolonged submergence, whereas the quiescence strategy is favored to withstand deeper and short-term submergence (Bailey-Serres and Voesenek, 2008; Manzur et al., 2009; Striker et al., 2017). The “escape strategy” syndrome includes fast elongation of shoot organs (Sauter et al., 1993; Müller et al., 2019) or leaf petioles (Groeneveld and Voesenek, 2003; Pierik et al., 2009) to restore air contact rapidly, enhanced adventitious root formation to improve oxygen uptake in water (Ayi et al., 2016; Zhang et al., 2017), and increased formation of internal aerenchyma tissue to efficiently transport gas (Manzur et al., 2009; Striker et al., 2019). These morph-physiological behaviors require carbohydrates for cell division and new cell production (Sauter, 2000; Voesenek et al., 2006; Luo et al., 2011). Therefore, one side effect of the “escape strategy” would be fast carbohydrate depletion (Bailey-Serres and Voesenek, 2008; Manzur et al., 2009; Akman et al., 2012), which is lethal for submerged terrestrial plants if they cannot protrude from the water surface. Under deep submergence conditions (where water depths are usually deeper than 2 m and light climate is poor), plants with a quiescence strategy will be more successful. They suppress energy consumption by only running basic metabolism with little or no organ elongation and new tissue formation, thus conserving carbohydrate reserves to prolong survival time in deep water while waiting for the water level to recede (Manzur et al., 2009; Pierik et al., 2009; Luo et al., 2011; Akman et al., 2012; Striker et al., 2017). Theoretically, it is likely that plants would survive and distribute from low to high elevations in the river riparian zones (or the reservoir drawdown zones and other flood disturbed habitats) if they are able to alter their response strategy at different submergence depths. It was reported that the wetland plant *Lotus tenuis* chose to escape from partial submergence by shoot elongation but adopted a non-elongating quiescent strategy when completely immersed in shallow water (Manzur et al., 2009). Nevertheless, the literature on whether terrestrial plants can alter their response strategies along a gradient of submergence depths is still scarce.

Besides gas and light, which induce plant responses to submergence, submergence depth—especially extremely deep submergence—may strongly affect plant performance (Vervuren et al., 2003). Submergence depth is a primary physical factor that varies along elevational gradients in many riparian regions (Howard and Mendelssohn, 1995). The effects of submergence depth on plant metabolism and growth can be direct or indirect (increasing hydrostatic pressure, increasing soil oxygen consumption, and changing temperature, which indirectly affects plant performance in water) (Grace, 1989; Howard and Mendelssohn, 1995; Casanova and Brock, 2000; Bejarano et al., 2018; Meng et al., 2022). Some studies have demonstrated the negative effect of increased submergence depth on plant survival; the median lethal time (LT_50_) of Rhine riparian plants at a depth of 1.6 m was approximately half of that at 0.4 m (Vervuren et al., 2003), similar to the effects observed in rice cultivars (Adkins et al., 1990). So far, the published literatures primarily reported the studies focused on the influences of submergence depths <2 m on terrestrial plants. The absence of studies investigating how deeper water affects plant performance might limit our understanding of plant distribution patterns in flood-prone habitats.

*Alternanthera philoxeroides* (Mart.) Griseb., a terrestrial perennial herbaceous plant belonging to the Amaranthaceae family, originates from South America and has spread to many parts of the world. It is considered an invasive species in the United States, Australia, New Zealand, Thailand, and China, and able to survive nicely in flood-disturbed habitats (Zhang et al., 2015a; Dong et al., 2018). *A. philoxeroides* exhibits an escape strategy in shallow submergence (Luo et al., 2009, 2011; Ayi et al., 2016), with quickly elongated shoots, increased adventitious roots formation, and widened aerenchyma channels conducive to enhance gas transport (Ayi et al., 2019). In addition, the species lowers its metabolic rate to substantially reduce carbohydrate consumption in water (Ye et al., 2016). *A. philoxeroides* not only distributes in shallow wetlands but also well exists in areas experiencing submergence with a maximum depth of 20 m for up to 4 months (Zheng et al., 2021). Therefore, this species is an ideal species for investigating how plants respond to submergence depth gradient. Considering the side effects of escape strategy and the wide distribution of *A. philoxeroides* in areas with varied submergence depths, we hypothesize that *A. philoxeroides* is likely to change its growth strategy when submergence depth differs.

In this study, we aimed to explore how *A. philoxeroides* plants respond to a gradient of submergence depths in terms of morphology, anatomy, biomass, and carbohydrate metabolism by conducting submergence experiments with varied water depths. Physical conditions of the water body including light, dissolved oxygen, pH, and temperature were kept constant in the experiments. The study may help understand the mechanisms of plant tolerance to extreme flooding and explain why *A. philoxeroides* remains highly invasive in regions where submergence depths are varied (e.g., the drawdown zones of large reservoirs). The findings may also provide insight into the effective management of this species.

## Materials and Methods

### Plant Material and Cultivation

*Alternanthera philoxeroides* is a herbaceous perennial plant, under normal conditions, it can spread quickly *via* clonal growth (Luo et al., 2009; Ayi et al., 2016), growing to a height of 50–120 cm, with a long single or sparsely branched stem. The stem has several internodes of different lengths, and its maturity degree gradually decreases from the base to the top, making it an ideal organ type to study the adaptation and response of *A. philoxeroides* to different submergence depths.

To reduce the influence of environmental conditions on mother plants, *A. philoxeroides* plants used in this experiment were cultivated from cuttings obtained from plants naturally growing on the banks of the Jialing River in Chongqing, Southwest China (29°49′N, 106°25′E). In May 2020, unbranched plants with a stem length of ~30 cm were selected and cut at the stem base. The cuttings were transported to the laboratory immediately, and healthy and vigorous cuttings were selected for subsequent treatments, all trimmed to have five internodes and six leaves. Each selected cutting was planted in a plastic pot (diameter and depth were both 13 cm) containing riparian soil from the Jialing River banks, two stem nodes of the cutting (hereafter referred to as “plant”) were buried in soil for rooting. All plants were cultivated under the same conditions and placed in an open field of the experimental garden affiliated to the Key Laboratory of Eco-environments in Three Gorges Reservoir Region (Ministry of Education) at Southwest University, Chongqing. The temperature, relative humidity, daily maximum light (PAR) intensity, and water provision were maintained at 10–15°C, 75–85%, 600–800 μmol m^−2^ s^−1^, and ~80–90% of soil water-holding capacity, respectively. Plants were watered daily. Lateral buds, if produced, were removed to ensure that the main stem of plants grew without branching. Plants were kept growing upright by the support of thin bamboo sticks. After ~1 month of cultivation, plants with ~45 cm in height and 12 internodes were selected for submergence treatments.

### Submergence Treatments

**Figure 1:**
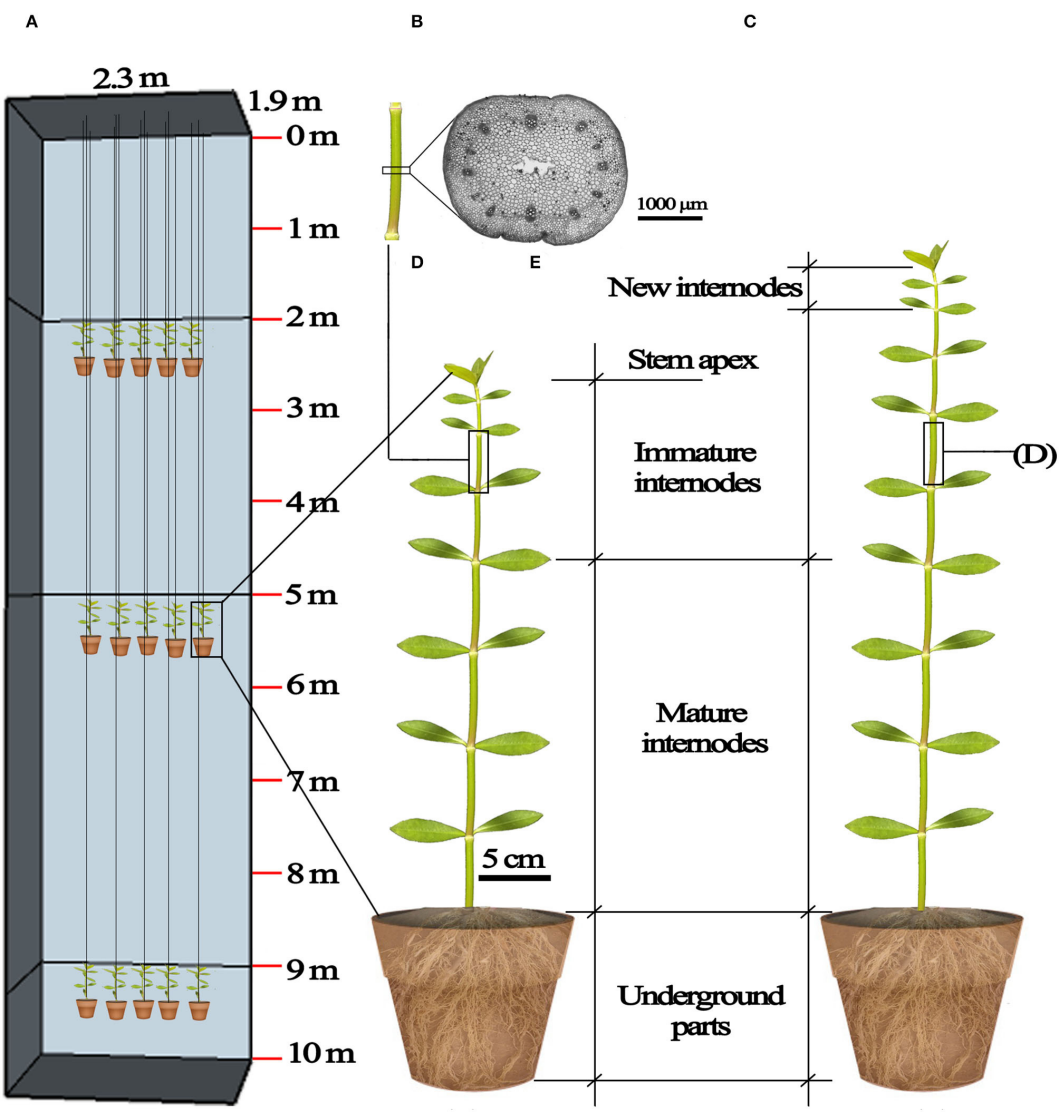
Diagrammatic representation of the submergence design and plant material. **(A)** The frame of the 10 m deep concrete reservoir (length × width × depth= 2.3 m ×1.9 m ×10 m) and the 2, 5, and 9 m submergence treatments; **(B)** underground parts, mature internodes, immature internodes and stem apex of *A. philoxeroides* before submergence treatments; **(C)** underground parts, mature internodes, immature internodes, new internodes, of *A. philoxeroides* after submergence; **(D)** the middle part of an internode, marked before treatments and used for making transverse sections at the end of submergence; **(E)** a transverse section of the internode.

Four submergence treatments were applied in a fully randomized design using selected plants (20 replicates per treatment). Submergence depth of 0 m was set as control: unsubmerged plants were placed under dark conditions and watered normally to keep soil water at field capacity. Additionally, three groups of plants were submerged in a water-filled concrete reservoir (length × width × depth = 2.3 × 1.9 × 10 m), with the top of plants 2, 5, and 9 m beneath the water surface. The plants in pots were suspended at planned water depths (Figure 1A). Pilot experiments showed that the stem tips of plants started to die (characterized by becoming flaccid) on the 7th day following submergence at depth of 9 m. Therefore, the treatment duration was set at six days for all submergence depths to ensure tested plants kept vigorous during this period.

### Water Environment Management

To investigate the effects of submergence depth on plants, the physico-chemical status of water (light, dissolved oxygen (DO), pH, and temperature) in the concrete reservoir were kept constant at any depths. The water was air-saturated by pumping air every day through an air pipe with vent holes installed at the bottom of the reservoir, ensuring the adequate and uniform supply of oxygen and carbon dioxide at various water depths. The temperature of the whole water body was kept constant by applying an electric heating system equipped at the bottom of the reservoir. The reservoir was covered with black sun-shading nets to eliminate the influence of light. The control treatment was conducted in darkness under the same temperature as the submergence treatments. DO concentration, photosynthetically active radiation (PAR) intensity, temperature, and pH of the water column at different depths in the reservoir were checked twice per day (morning and evening) using a multi-parameter water quality analyzer (Hydrolab DS5, Hach, United States). During the experiment, no significant difference in these factors was found among different water depths (Table 1).

**Table 1:** Physico-chemical properties of water in submergence reservoir.

**Submergence depth** **(m)**	**Dissolved oxygen concentration (mg L^**−1**^)**	**Temperature (**°**C)**	**pH**	**PAR (μmol m^**−2**^ s^**−1**^)**
0	n.a.	23.49 ± 0.13 a	n.a.	0 a
2	9.21 ± 0.16 a	23.45 ± 0.05 a	7.02 ± 0.02 a	0 a
5	9.19 ± 0.18 a	23.38 ± 0.05 a	7.03 ± 0.01 a	0 a
9	9.12 ± 0.09 a	23.28 ± 0.07 a	7.04 ± 0.00 a	0 a

*The dissolved oxygen, temperature, photosynthetically active radiation (PAR), and pH of the water were checked at different depths twice per day (in the morning and evening) using a multi-parameter water quality analyzer (Hydrolab DS5, Hach, USA) during the experiments (mean ± S.E.; n = 13); n.a. indicates no data. Same lower-case letter indicates no significant difference (p > 0.05) between submergence depths*.

### Growth Measurements

Each plant had ~12 stem internodes at the start of treatments. From the stem base upwards, the 1st to 6th internodes were mature and the 7th to 12th internodes were immature (Figure 1B). We marked non-destructively immature internodes so as to distinguish between the mature, immature internodes formed before treatment and the new internodes produced during treatment. At the beginning of treatments, for each plant, 26 leaves were retained on the upper stem by trimming other leaves from the stem base upwards. The length of all internodes of each plant was measured twice (at the beginning and the end of treatments). We compared the elongation of mature, immature, and newly produced internodes at the end of the treatments to assess the effects of submergence depths on the growth of internodes of varying maturity degrees. The elongation of mature internodes, immature internodes, and newly produced internodes was, respectively, calculated as the sum of elongation from the 1st to 6th internodes, the sum of elongation from the 7th to 12th internodes, and the length sum of all newly produced internodes (Figure 1C). The number of newly produced internodes was also counted. Afterward, the leaves, stem (viz. the aboveground stem part), and underground part (including the underground stem part which can be regarded as rhizome, and roots) of each plant were oven-dried to constant weights at 75°C, and their dry mass was determined (BSA124S, Sartorius, Germany).

No adventitious roots were formed on the aboveground stem nodes of *A. philoxeroides* before the submergence treatments. Any formation of aquatic adventitious roots on the aboveground stem nodes was recorded at the end of the treatments.

### Transverse Section and Pith Cavity of Internodes

Because the pilot experiment showed that the 10th stem internode had relatively large elongation during treatments (Supplementary Figure S1), and its pith cavity had not been formed at the start of the treatments, we marked the 10th internode before treatments to investigate the effects of submergence depths on the development of internodal pith cavity. Transverse sections were made at the middle of the internodes immediately when treatments terminated (Figures 1D,E). Sections were observed and photographed using a stereomicroscope (SMZ25, Nikon, Japan). The pith cavity cross-area of each section was measured using the NIS-elements imaging software (version 4.30).

### Non-Structural Carbohydrate Analysis

Soluble sugars and starch of the dried stems and underground parts of plants were measured by using fine powder samples prepared with a ball mill (WS-MM200, Retsch, Haan, Germany). Soluble sugars and starch were extracted and determined using a modified method based on the traditional anthrone-sulfuric acid method (Zhang, 2003; Lei et al., 2014). Ethanol-soluble sugars of 0.01 g powder sample soaked in 80% (v/v) ethanol solution were extracted in the water bath at 80°C for 40 min. The extraction of each sample was repeated twice and the extracts were mixed and diluted with ultrapure water to 50 ml. Subsequently, the residue of each sample was soaked in 5 ml ultrapure water and extracted for soluble but ethanol-insoluble sugars in the water bath at 80°C for 40 min, the extraction of each sample was also repeated twice and the extracts were mixed and diluted with ultrapure water to 50 ml. The starch of the residue was hydrolyzed to soluble sugars using 6 mol L^−1^ HCl, the hydrolyte solution was filtered into a flask and diluted with ultrapure water to 100 ml. A total of 1 ml of the above-mentioned 50, 50, and 100 ml diluted extracts was respectively added with 5 ml anthrone-sulfuric acid reagent (0.1 g anthrone dissolved in 100 ml 75% (v/v) sulfuric acid solution) and heated for 10 min at 100°C, and the light absorbance at 625 nm was measured (UV-2700, Shimadzu, Japan) to determine the concentration of ethanol-soluble sugars, soluble but ethanol-insoluble sugars, and starch based on a glucose calibration curve. The concentration of total soluble sugars in a plant sample was the concentration sum of ethanol-soluble sugars plus soluble but ethanol-insoluble sugars. Since the starch was hydrolyzed to soluble sugars, starch concentration was therefore calculated by multiplying the concentration of hydrolytic soluble sugars with a hydrolysis coefficient (0.9). The sum of ethanol-soluble sugars, soluble but ethanol-insoluble sugars, and starch was regarded as non-structural carbohydrates in plant samples.

### Data Analyses

The effects of treatments (control and submergence at different depths) on stem and internode elongation, plant and organ biomass, number and length of newly produced internodes, internodal pith cavity cross area, number of nodes forming adventitious roots, and contents of non-structural carbohydrates (soluble sugars and starch) were checked using one-way ANOVA. Logarithm transformation of data was performed to equalize variance if necessary. Differences between treatments were detected using Duncan's multiple range test, and the significance level was set at *p* = 0.05. All the analyses were conducted using SPSS 22 (SPSS Inc., Chicago).

## Results

### Stem Elongation and new Internode Production

**Figure 2:**
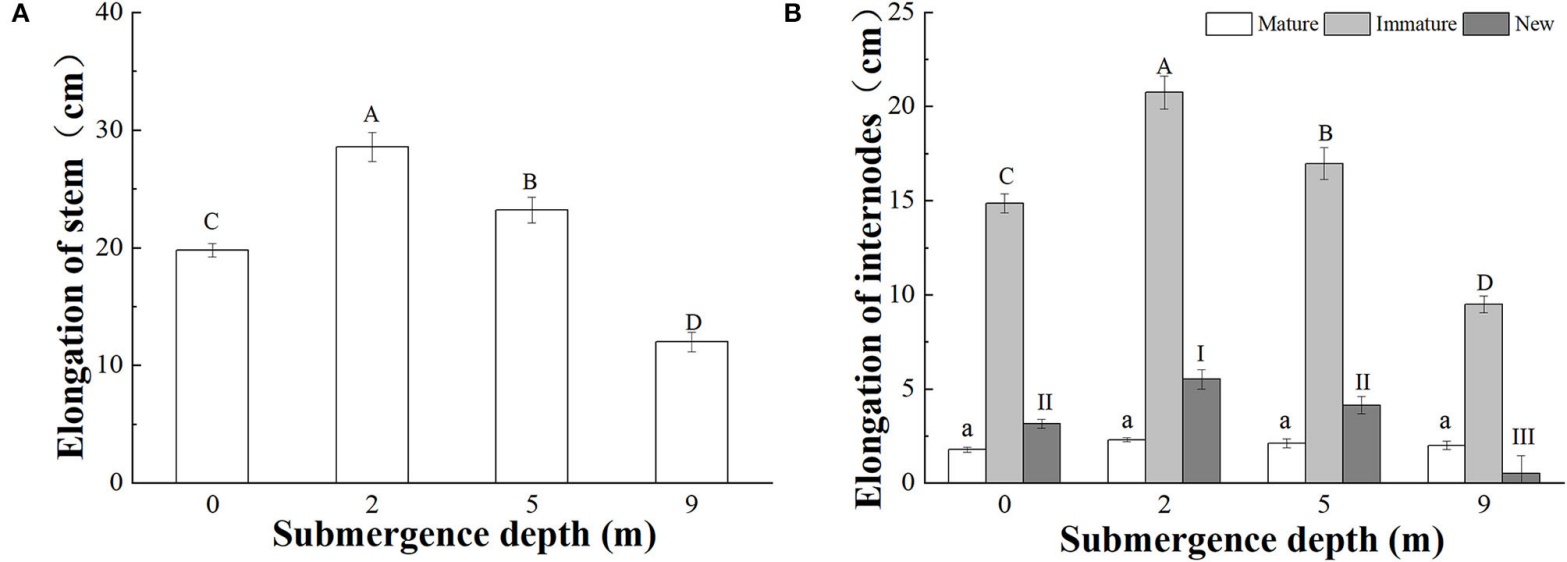
Stem elongation of **(A)** the whole stem; **(B)** mature and immature internodes before the treatments, and new internodes produced during the treatments, of *A. philoxeroides* at the end of treatments at different submergence depths (mean ± S.E; *n* = 20). Different lower-case letters indicate a significant difference (*p* < 0.05) in the elongation of mature internodes between treatments. Different upper-case letters indicate significant differences (*p* < 0.05) in elongation of the whole stem **(A)** and immature internodes **(B)** between treatments. Different roman letters indicate significant differences (*p* < 0.05) in the elongation of new internodes between treatments.

*A. philoxeroides* plants subjected to four treatments [water depths of 0 m (control), 2, 5, and 9 m] all elongated their stems during the experiment; however, stem elongation significantly decreased with increasing submergence depth after 6 days of treatment (Figure 2A). The stem elongation of plants subjected to submergence at a water depth of 0 (control), 2, 5, and 9 was 19.79, 28.56, 23.21, and 11.99 cm, respectively. Plants submerged at water depths of 2 and 5 m presented significantly larger stem elongation than control plants, but plants submerged at water depth of 9 m presented much less stem elongation than control plants (Figure 2A). The contribution of mature and immature internodes produced before treatments and new internodes produced during treatment to the stem elongation differed significantly, immature internodes comparatively made the largest contribution to plant stem elongation (Figure 2B).

The elongation responses to submergence depths differed between mature, immature, and newly produced internodes. The total elongation of mature internodes was not affected by submergence depths, but the total elongation of immature internodes and newly produced internodes differed significantly between submergence depths (Figure 2B). The total elongation of immature internodes were 14.86, 20.75, 16.95, and 9.48 cm in plants submerged at water depths of 0 (control), 2, 5, and 9 m, respectively (Figure 2B).

Very few new internodes were produced during submergence at water depth of 9 m, and 1.85, 2.40, and 1.85 new internodes on average were produced at water depths of 0, 2, and 5 m, respectively (Table 2). The total length of new internodes was significantly different between the treatments, with the largest length realized in plants submerged at depth of 2 m (Figure 2B).

**Table 2:** Internode numbers at the start and the end of treatments (means ± S.E. *n* = 20).

**Submergence depth (m)**	**Internode numbers**		**New internode numbers**
	**Before submergence**	**After submergence**	
0	12.50 ± 0.21a	14.40 ± 0.22a	1.85 ± 0.08b
2	12.30 ± 0.25a	14.60 ± 0.29a	2.40 ± 0.11a
5	12.20 ± 0.25a	14.00 ± 0.25a	1.85 ± 0.13b
9	12.40 ± 0.22a	12.75 ± 0.27b	0.30 ± 0.11c

*Different lower-case letters indicate significant differences (p < 0.05) between submergence depths*.

Based on the results of this study, it was found that the stem elongation and internode production of submerged *A. philoxeroides* decreased with increasing submergence depths, with the largest stem elongation and new internode production showing under the shallowest submergence (2 m depth) and the smallest stem elongation and nearly no new internode production under the deepest submergence (9 m depth). However, in comparison with plants unsubmerged, the stem elongation and internode production of plants were enhanced by submergence at depths of 2 and 5 m.

### Internodal Pith Cavity and Adventitious Root Formation

**Figure 3:**
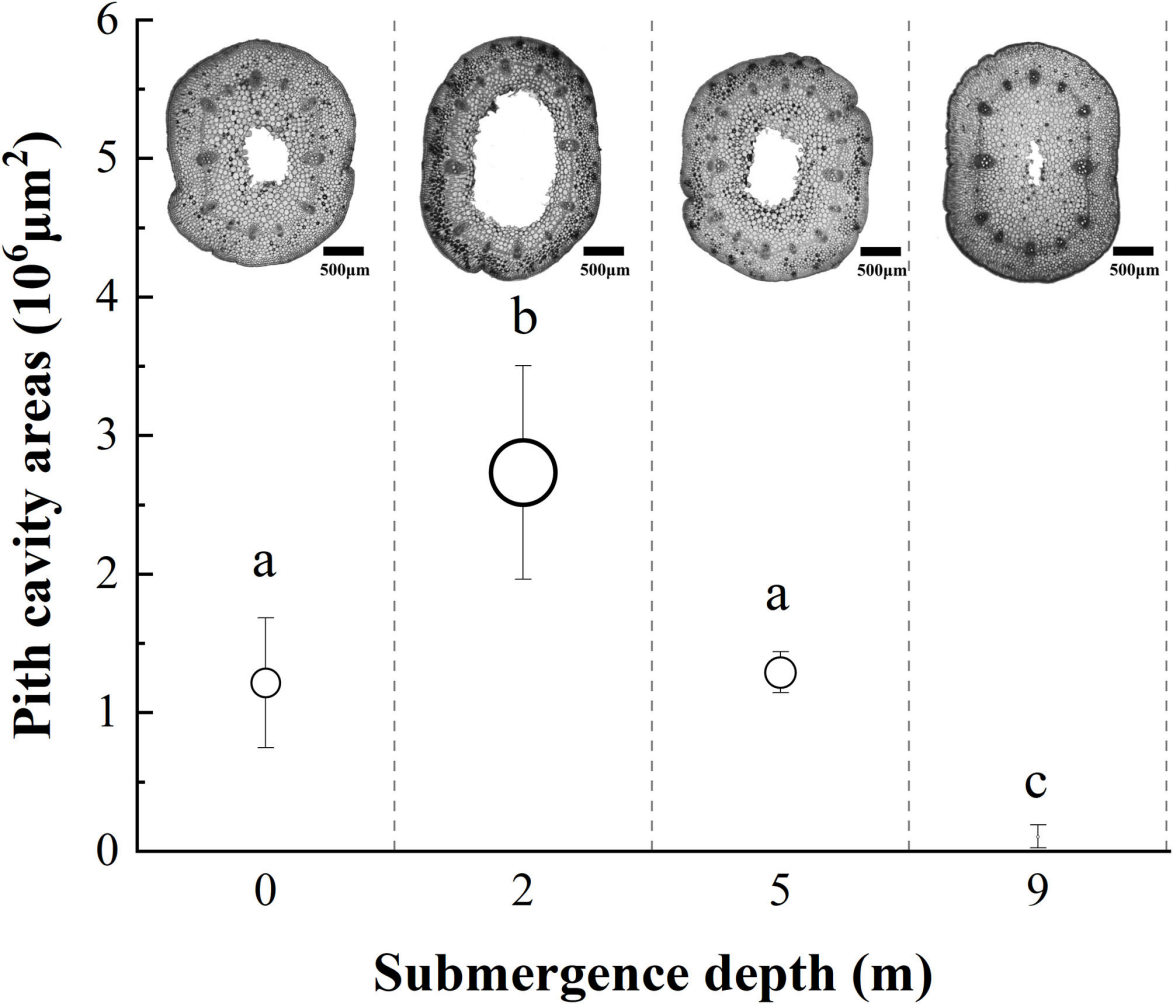
The pith cavity transverse areas of immature internodes of *A. philoxeroides* at the end of treatment at different submergence depths (mean ± S.E; *n* = 3). Sections were made at the middle position of internodes. Different lower-case letters indicate significant differences (*p* < 0.05 using one-way ANOVA) between treatments.

The internodal pith cavity developed during the experiment varied among four treatments (Figure 3). Plants submerged at depths of 2 and 9 m had the broadest and the narrowest internodal pith cavity, respectively. Plants submerged at depth of 5 m did not differ from unsubmerged plants in size of internodal pith cavity (Figure 3).

**Figure 4:**
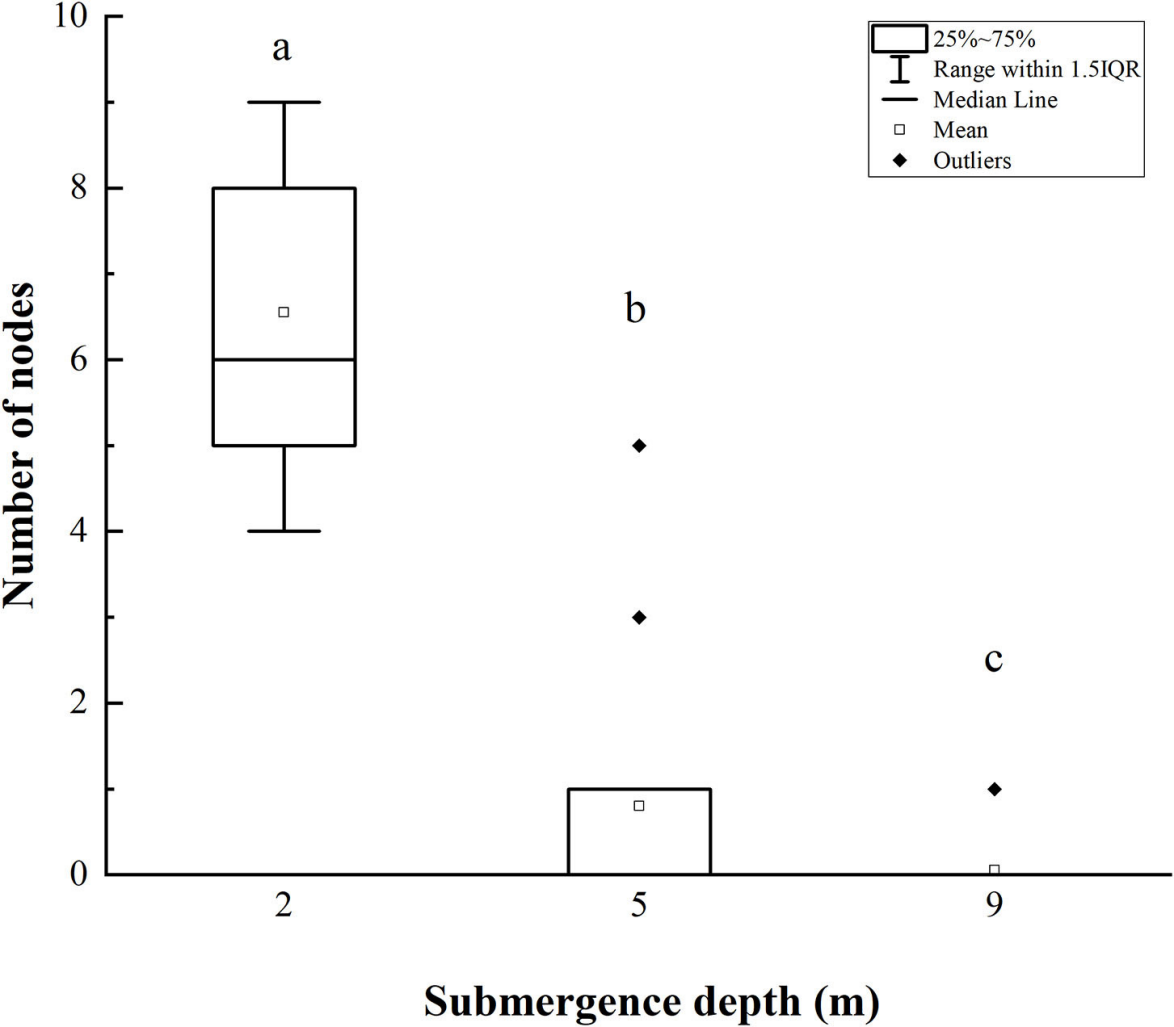
The number of stem nodes forming adventitious roots of *A. philoxeroides* at the end of submergence treatments (mean ± S.E.; *n* = 20). Different lower-case letters indicate significant differences (*p* < 0.05 using one-way ANOVA) between treatments.

The number of stem nodes forming adventitious roots upon submergence decreased significantly with increasing submergence depth (Figure 4). Approximately 4–9 nodes (an average of 6.5) on plants submerged at depth of 2 m formed adventitious roots, and 0–5 nodes (an average of 1) on plants at a submergence depth of 5 m formed adventitious roots, only 1 node of 1 plant (20 plants in total) formed adventitious roots when submerged at depth of 9 m (Figure 4).

It was obvious that increasing water depth impeded the pith cavity development and adventitious root formation of submerged *A. philoxeroides* plants.

### Biomass and Carbohydrates

**Figure 5:**
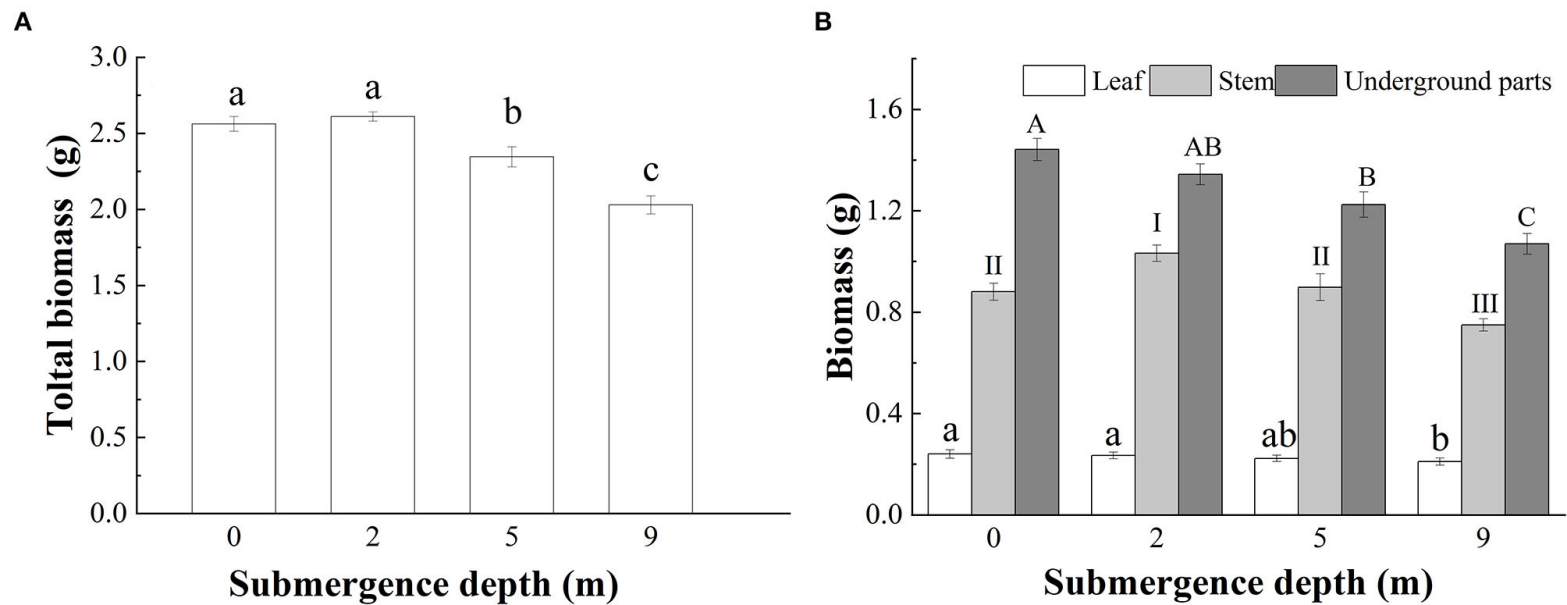
Biomass of **(A)** whole plant; and **(B)** leaves, stems, and underground parts of *A. philoxeroides* at the end of submergence treatments (Mean ± S.E, *n* = 20). Different lower-case letters indicate significant differences (*p* < 0.05) in the total plant mass **(A)** and the leaf mass **(B)** between treatments, different roman letters, and upper-case letters, respectively indicate significant differences (*p* < 0.05) in stem mass and underground plant mass between treatments.

At the end of the experiment, the total biomass of submerged *A. philoxeroides* plants decreased gradually with increasing submergence depth, however, unsubmerged plants and plants submerged at a depth 2 m did not differ in total biomass (Figure 5A). The total leaf mass of plants submerged at a depth 9 m was the smallest among all treatments, but the total leaf mass did not differ between plants submerged at depths of 2 and 5 m, and plants unsubmerged. The largest and the smallest stem mass presented respectively in plants submerged at depths of 2 and 9 m, and plants submerged at depth of 5 m had similar stem mass to control plants (Figure 5B). Unlike the responses of stem and total leaf mass to treatments, the underground mass of plants declined with increasing submergence depths, plants unsubmerged tended to achieve the largest underground mass, and plants submerged at a depth 9 m had the smallest underground mass (Figure 5B).

**Figure 6:**
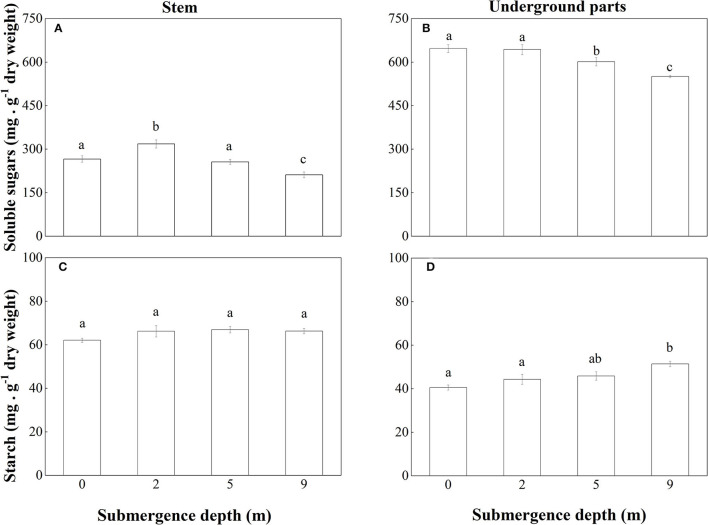
Concentrations of soluble sugars in **(A)** stems and **(B)** underground parts; starch in **(C)** stems and **(D)** underground parts of *A. philoxeroides* at the end of the submergence treatments (Mean ± S.E; *n* = 5). Different lower-case letters indicate significant differences (*p* < 0.05 using one-way ANOVA) between treatments.

The non-structural carbohydrates of *A. philoxeroides* plants were mainly composed of soluble sugars. At the end of the experiment, the concentration of soluble sugars in both stems and underground parts of submerged plants decreased with increasing submergence depth (Figures 6A,B). As to the concentration of soluble sugars in stems, unsubmerged plants were not different from plants submerged at depth of 5 m but were lower than plants submerged at depth of 2 m and higher than plants submerged at depth of 9 m (Figure 6A). Unsubmerged plants were similar to plants submerged at depth of 2 m in the concentration of soluble sugars in the underground plant parts but had a higher concentration of soluble sugars than plants submerged at depth of 5 and 9 m (Figure 6B).

No significant difference was found between treatments in starch concentration in stems of *A. philoxeroides* (Figure 6C). But starch concentration in underground plant parts tended to increase with increasing submergence depth, plants submerged at depth of 9 m had the highest concentration of starch (Figure 6D).

## Discussion

This study aimed to investigate the responses of terrestrial plants to alteration in submergence depth. In some previous studies, the escape or quiescence strategies selected by submerged plants were extensively discussed (Bailey-Serres and Voesenek, 2008; Voesenek and Bailey-Serres, 2015). The strategy selection by plant species was directly linked to where the species is able to distribute in habitats prone to flooding at various depths (Parolin, 2002). However, whether species having wide distribution in flood-prone habitats can alter their response strategy to cope with various submergence depths is rarely discussed. Using *A. philoxeroides* as a model species, this study demonstrated that strategy change as a response to submergence depths alteration was possible in flood-tolerant plants.

### Escape Strategy at Submergence Depth of 2 m

*A. philoxeroides* exhibited a typical “escape strategy” when submerged at depth of 2 m *via* fast shoot elongation by producing new internodes and elongating its immature internodes (Figure 2, Supplementary Figure S1, Table 2). Although previous studies showed that escape strategy was mainly presented in plants submerged partially or in waters shallower than 2 m depth (Bailey-Serres and Voesenek, 2008; Manzur et al., 2009; Akman et al., 2012), our present work indicated that escape strategy can also be selected by *A. philoxeroides* under submergence depths equal or larger than 2 m, which implies that *A. philoxeroides* can escape from the complete submergence with a water depth of 2 m or even deeper. Theoretically, the fast elongation of stems under submergence at depth of 2 m might be lethal to plants because of the substantial energy consumption and consequent energy crisis. However, our experimental results showed that the biomass and carbohydrate consumption of plants at 2 m submergence depth did not differ from those of unsubmerged plants at the end of the experiment (Figures 5, 6). This might be due to the morpho-physiological behaviors the plant took to help mitigate possible damages, especially energy crisis. First, adventitious root formation was enhanced in *A. philoxeroides* plants submerged at depth of 2 m (Figure 4), which was beneficial for plants to improve the supply of oxygen and mineral nutrients under submergence stress, because adventitious roots are able to absorb oxygen and mineral nutrients from water (Ayi et al., 2016, 2019). Second, the plants submerged at depth of 2 m developed the widest internodal pith cavity (Figure 3), which surely facilitated plants' internal gas transport (Jackson and Armstrong, 1999; Pedersen et al., 2021). Therefore, our results indicated that *A. philoxeroides* was able to resist submergence of 2 m depth by adopting an escape strategy *via* stem elongation, pith cavity development, and adventitious roots formation.

### Quiescence Strategy at Submergence Depth of 9 m

When submerged at depth of 9 m, *A. philoxeroides* adopted a quiescence strategy by minimizing its growth, including almost no new internode production (Table 2) and new internode elongation (Figure 2), suppressed pith cavity development (Figure 3) and adventitious root formation (Figure 4). This quiescence had a “two-edged sword” effect. On one hand, the weakened growth of new tissues and organs led to low consumption of substances including carbohydrates; on the other hand, suppressed pith cavity development and adventitious roots formation decreased the utilization efficiency of carbohydrates under deep submergence conditions. The former would reduce the carbohydrate requirement of submerged plants and enhance their submergence tolerance; the latter may lead to fast carbohydrate consumption, which would be lethal under prolonged submergence. Our results showed that the loss of total biomass in plants submerged at depth of 9 m was significantly higher than that in unsubmerged plants (Figure 5), and the concentration of soluble sugars was lower in plants submerged at depth of 9 m than in unsubmerged plants (Figures 6A,B). Nevertheless, all plants submerged at depth of 9 m survived with no tissue corruption, indicating the high submergence tolerance of *A. philoxeroides*.

### Strategy Shift at Intermediate Submergence Depth

The responses of *A. philoxeroides* to submergence at depth of 5 m indicated that the plant was probably in the process of strategy shift from escape to quiescence. Plants submerged at depth of 5 m presented larger stem elongation than unsubmerged controls (Figure 2), which was an obvious sign of escape strategy. However, the reduced adventitious root formation, decreased total plant biomass, and lowered soluble sugars concentration in the underground parts of plants submerged at depth of 5 m (Figures 4–6), as compared to those of unsubmerged controls, revealed quiescence strategy was also adopted by plants submerged at depth of 5 m. Thus, we inferred that *A. philoxeroides* may gradually switch its strategy from “escape” to “quiescence” as a response to submergence with increasing depth. It was reported that *Lotus tenuis* could quickly switch from an escape strategy under partial submergence to a quiescence strategy when confronted with complete shallow submergence (Manzur et al., 2009). Obviously, *A. philoxeroides* did not take this quick strategy switchover but performed gradual change in strategy when submergence depth altered.

Previous studies have pointed out that *A. philoxeroides* is a representative species that adopts an escape strategy in response to submergence (Luo et al., 2009, 2011; Ayi et al., 2016). This study supported this argument. However, it was also found in this study that *A. philoxeroides* was able to change its strategy from “escape” to “quiescence” under deep submergence (deeper than 5 m in this study), an intriguing phenomenon that was not observed in previous studies. Based on our experimental results, two questions need to be more focused on in the following studies are raised: (1) do all species that typically adopt escape strategy automatically alter their strategy with increasing submergence depth and (2) what role does the submergence depth play in the strategy switchover?

### Role of Hydrostatic Pressure

Low light, whether in terrestrial or in aquatic habitats, is an inducing factor for plants to adopt an escape strategy (via fast shoot and petiole elongation) due to shade avoidance (Mommer et al., 2005; Pierik et al., 2011; Sasidharan et al., 2014). In this study, the stem elongation of *A. philoxeroides* submerged at depths of 0, 2, and 5 m in darkness substantiated this statement (Table 2; Figure 2); however, the retarded growth of *A. philoxeroides* submerged at depth of 9 m in the same darkness (Table 2; Figure 2) suggested that the light availability can not sufficiently explain the strategy change along a gradient of submergence depths. If the stem elongation upon submergence were induced by low light or darkness due to phototaxis of plants, plants submerged at any depths in darkness should present similar stem elongation.

Generally, the escape and quiescence strategies represent the syndrome that plants exhibit as a response to low oxygen levels in water (Voesenek and Bailey-Serres, 2013, 2015). Oxygen shortage might induce the production of reactive oxygen species (Paradiso et al., 2016; Sasidharan et al., 2018), nitric oxide (Mugnai et al., 2012; Paradiso et al., 2016), ethylene (Fukao and Bailey-Serres, 2008; Sasidharan et al., 2018), and other signaling molecules (Sasidharan et al., 2018), as well as activate a series of phytohormone-synthesizing molecules (Cox et al., 2004) to induce different strategies among species. In plant species adopting an escape strategy, the low oxygen level not only stimulates the elongation of shoots and petioles, but also induces aerenchyma formation, *via* increasing porosity in the roots or stem (Parlanti et al., 2011; Pedersen et al., 2021), widening the pith cavity (Steffens et al., 2011), and forming adventitious roots (Zhang et al., 2015b; Ayi et al., 2016). However, in a water body with dissolved oxygen saturated (like the water environment *A. philoxeroides* experienced in this study), it is hard to attribute the strategy shift of plants submerged at different water depths to oxygen availability.

In our experimental system, all factors except water depth were kept constant (Table 1). Therefore, it is logical to infer that water depth was linked to the strategy changeover of *A. philoxeroides*. It was found in previous studies that water depth affected plant distribution *via* hydrostatic pressure (Dale, 1981; Makarov, 2011). Some studies suggested that low hydrostatic pressure could accelerate physiological activities and stimulate plant growth, whereas high hydrostatic pressure may affect the stabilization of cells or enzymes, depress physiological activities, and impede cell division or elongation (Adkins et al., 1990; Vervuren et al., 2003; Yi et al., 2016; Bejarano et al., 2018). The strategy changeover of *A. philoxeroides* along submergence depth gradient in this study was very probably caused by the change in hydrostatic pressure, but how hydrostatic pressure induces strategy changeover needs to be clarified.

## Conclusion

This study revealed that submerged *A. philoxeroides* can gradually change its response strategy from “escape strategy” to “quiescence strategy” when water depth was increasing. Notably, this changeover took place under deep submergence (deeper than 5 m), which implies that water depth plays an important role in the strategy selection of plants in response to submergence. According to our observations, we found the morphological responses of *A. philoxeroides* to submergence of different depths were chiefly the outcome of acclimatization, because the plants presenting strong stem elongation under shallow submergence did not exhibit stem elongation when transferred to deep submergence, and plants presenting no stem elongation under deep submergence can still show strong stem elongation upon shallow submergence. Undoubtedly, this acclimatization enhances greatly the capability of *A. philoxeroides* in coping with floods with unpredictable depths. Further studies should focus on understanding the driving mechanism of strategy changeover of plants submerged at different water depths, especially the driving effects of hydrostatic pressure.

## Data Availability Statement

The original contributions presented in the study are included in the article/Supplementary Material, further inquiries can be directed to the corresponding author/s.

## Author Contributions

BZ and XZ conceived the original research plan, designed, and supervised the experiments. SJ and HN performed most of the experiments. BW, XR, XS, and SS provided assistance for some experiments. SJ, XZ, QA, and BZ analyzed the data and wrote the article with contributions from SL and FL. All authors contributed to the article and approved the submitted version.

## Funding

This work was supported by the National Natural Science Foundation of China (Grant Numbers 31400480, 31800331, and 31770465) and Chongqing Talents Program (Grant Number cstc2021ycjh-bgzxm0316).

## Conflict of Interest

The authors declare that the research was conducted in the absence of any commercial or financial relationships that could be construed as a potential conflict of interest.

## Publisher's Note

All claims expressed in this article are solely those of the authors and do not necessarily represent those of their affiliated organizations, or those of the publisher, the editors and the reviewers. Any product that may be evaluated in this article, or claim that may be made by its manufacturer, is not guaranteed or endorsed by the publisher.
