# Supplementary material for: Differential Growth Responses of Alternanthera philoxeroides as Affected by Submergence Depths
Source: Front Plant Sci. 2022 Jun 2;13:883800. doi: 10.3389/fpls.2022.883800 (PMC9201830; doi:10.3389/fpls.2022.883800)
Supplement: Supplementary file 1 [file Data_Sheet_1.docx]

Supplementary Material

## Supplementary Figure

Supplementary Figure 1 Elongation of every internode which formed before treatments of *A. philoxeroides* at the end of treatments (Mean, n = 20). The 1st to 12th internode of plants is count from the base of the plant before submerged. The 1st to 6th internode were mature internodes, and the 7th to 12th internode were the immature internodes.
